# A Prospective Observational Quality Improvement Study of a Two-Step Ultrasound Protocol for Guidewire Confirmation During the Internal Jugular Central Venous Catheter Placement

**DOI:** 10.7759/cureus.92911

**Published:** 2025-09-22

**Authors:** Maria Nefeli Vouri Kokkori, Amanuel N Teklu, Nicole D Hilber, Konstantin Prosenz, Eckhard Mauermann

**Affiliations:** 1 Anesthesiology, Zurich City Hospital, Zurich, CHE; 2 Medical School, University of Basel, Basel, CHE

**Keywords:** anesthesia, central venous catheter, guidewire confirmation, internal jugular vein, malposition, quality improvement, ultrasound

## Abstract

Background

Malposition of central venous catheters (CVCs) remains a relevant complication despite the use of ultrasound guidance. Traditional radiographic confirmation increases cost and radiation exposure. A minimal ultrasound protocol for guidewire confirmation may help reduce malposition rates without postprocedural radiographic imaging.

Objectives

To assess the effectiveness of a standardized two-step ultrasound protocol for confirming guidewire placement during internal jugular vein (IJV) CVC insertion in reducing catheter malposition rates.

Methods

This was a prospective observational quality improvement project conducted over six months in the anesthesia department of a tertiary hospital. Adult patients undergoing elective IJV CVC placement were included. A standardized two-step ultrasound protocol for guidewire confirmation during IJV CVC placement was introduced: (1) confirmation of caudal guidewire progression in the IJV and (2) exclusion of guidewire presence in the ipsilateral subclavian vein. Protocol adherence was evaluated prospectively by obtaining images and/or videos documenting the two-step ultrasound verification process. These recordings were captured by either the resident or the attending physician during the placement of the guidewire. All collected media were subsequently reviewed to confirm compliance with each step of the protocol. Catheter tip location was assessed routinely via postprocedural chest X-rays. The primary outcome was CVC malposition. Statistical analysis included Fisher’s exact test and descriptive statistics.

Results

Out of 524 procedures, 506 were analyzed, while 18 were excluded due to a lack of postprocedural chest X-rays. Complete protocol adherence occurred in 243 (48%) cases. No malpositions occurred in the protocol-adherent group (0/243; 0%, 95% confidence interval (CI): 0-1.2%), whereas seven malpositions occurred among 263 partially adherent and non-adherent cases (2.7%). Fisher’s exact test showed a significant association between non-adherence and malposition (p = 0.015). One contralateral pneumothorax unrelated to CVC insertion was observed.

Conclusion

A simple two-step ultrasound protocol for guidewire confirmation was associated with a significantly lower rate of CVC malposition. The method is feasible, efficient, and may serve as an alternative to routine radiographic confirmation in appropriate clinical contexts.

## Introduction

Scope and relevance of the study question

It is estimated that several million central venous catheters (CVCs) are placed annually [1]. These catheters are often essential for administering medication, fluids, and monitoring haemodynamic parameters. However, their placement is not without risks, and malpositioning occurs in a significant proportion of cases, depending on the definition [1,2]. While there is an obvious risk of pneumothorax [1,3] and inadvertent arterial placement [4,5], even malpositioning of catheters within the venous system bears potential risks. Particularly, catheter dysfunction and venous thrombosis for catheters in the subclavian vein have been described [6].

Despite the widespread adoption of ultrasound for CVC placement, there is no standardized protocol for its use in confirming the final catheter position. The correct position of the catheter tip and the absence of pneumothorax are typically visualized using routine post-procedural X-rays. However, this is resource and cost-intensive and exposes the patient unnecessarily to radiation. Alternatively, ultrasound may be utilized not only for the puncture itself [7-9] (e.g., with a linear or curved array probe), but also for excluding pneumothorax and for confirming catheter position using a transthoracic echocardiography (TTE) probe. Indeed, several studies have shown a point-of-care ultrasound (POCUS) TTE approach to be feasible and promising [10-12]. However, this is also time and resource-intensive.

We wondered whether a minimal ultrasound assessment, performed in real-time during CVC insertion, could be used to decrease the rate of incorrect CVC placement, despite not directly visualizing the wire tip or the catheter itself after placement. This protocol consisted of (1) confirming the position of the guidewires caudally in the internal jugular vein (IJV) and (2) excluding guidewires in the ipsilateral subclavian vein. Essentially, when using ultrasound to place a CVC, this mini protocol involves only two brief images or videos, thereby minimizing the incremental effort. While a direct visualization of the guidewire using a “notch view” from above the clavicle towards the heart with a curved array probe may be possible, this probe is not always available, and the view may be more suited for subclavian cannulation [5]. Our minimal approach is that any linear probe could be used.

The primary aim of this observational prospective quality control analysis was to determine the rate of CVC malposition when using our two-step ultrasound protocol compared with cases of partial adherence (only one step performed) or non-adherence (protocol not followed). Specifically, we hypothesized that there would be a less than 3% malposition rate using this method. Additionally, we sought to compare the rate of malposition when utilizing the mini protocol versus not utilizing it. As a safety endpoint, we also examined the rate of pneumothorax.

## Materials and methods

Study design and setting

We conducted a prospective observational quality improvement project over a six-month period (November 2024-April 2025) at the Department of Anesthesiology, Zurich City Hospital, Zurich, Switzerland. Our project evaluated the effectiveness of a standardized two-step ultrasound protocol for real-time confirmation of guidewire placement during IJV CVC insertion. The protocol was implemented as part of a departmental quality improvement program.

Ethics and regulatory considerations

As this was a quality improvement initiative embedded in routine clinical practice, the project was exempt from formal ethical review according to Swiss Human Research Act (HRA) regulations. All procedures adhered to institutional policies for patient safety, data confidentiality, and anonymized data handling.

Study population and eligibility

We included all adult patients (aged 18 years or older) undergoing CVC placement via the IJV performed by anesthesia personnel (residents and attending physicians as clinically performed) in the operating theatre or perioperative setting. Exclusion criteria comprised placements performed in the emergency department or intensive care unit (ICU), as these settings often involve time constraints or patient instability that could confound protocol adherence. Demographic data (e.g., age, sex) were not systematically collected, as all adult patients (≥18 years) undergoing elective IJV CVC placement during the study period were included without exclusion based on clinical characteristics.

Ultrasound protocol and procedural technique

The technique of CVC placement in the IJV was performed at the physician’s discretion. However, we recommended using our simple two-step ultrasound protocol. This protocol was designed to confirm proper catheter placement in real time. First, operators were required to dynamically track the guidewire in the IJV using ultrasound, following its caudal progression toward the superior vena cava (SVC) while recording a brief video clip. This step ensured visualization of the guidewire’s trajectory and excluded aberrant paths (e.g., retrograde cephalad). Second, providers performed a systematic examination of the ipsilateral subclavian vein, obtaining either an in-plane or out-of-plane view to document the absence of catheter malposition. Both steps were performed using Sonosite ultrasound machines (Fujifilm Sonosite, Bothell, USA) equipped with high-frequency linear transducers. Routine clinically mandated X-rays were examined after CVC placement.

To standardize the ultrasound protocol across providers, we conducted a 15-minute group presentation detailing the technique, followed by the distribution of laminated flashcards summarizing the key procedural steps. The pocket-sized flashcards served as quick-reference guides at the point of care, featuring schematic diagrams and criteria for adequate ultrasound documentation.

No additional simulation training or competency assessments were required, as all participating anesthesiologists had prior experience in ultrasound-guided vascular access. Protocol adherence was reinforced through regular reminders and the availability of the reference materials in all procedural areas. Post-procedural chest X-rays were performed per routine clinical practice to confirm catheter position and exclude pneumothorax.

Data collection and outcome measures

Data were prospectively collected for all CVC placements during the observation period. For each procedure, we documented whether the operator fully followed the ultrasound protocol (two documented ultrasound images or videos), partially adhered (e.g., performed only one step), or deviated entirely (e.g., no ultrasound images or used landmark technique without ultrasound confirmation). Post-procedural chest X-rays were reviewed by an independent radiologist blinded to the ultrasound findings. These X-rays were assessed for catheter tip position and any evidence of pneumothorax or other complications.

The primary outcome was CVC malposition, defined as the tip being in the subclavian vein, in the contralateral innominate vein, or retrograde cephalad in the IJV in the postprocedural X-ray. The secondary outcome was the presence of pneumothorax on post-procedural chest radiograph in patients without recent ipsilateral thoracic surgery.

Outcome assessment

All post-procedural chest radiographs were reviewed by an independent radiologist blinded to protocol adherence status.

Statistical analysis

Data are presented as median (Q1-Q3) or count (%), as appropriate. Event rates were calculated as the number of events divided by the total number of cases. The 95% confidence interval (CI) was determined using the log method: $CI = (\exp(\ln(r) - 1.96 \times SE), \exp(\ln(r) + 1.96 \times SE)), \text{ where } r = \frac{x}{n} \; (x = \text{number of events}, n = \text{number of cases}), \; SE = \sqrt{\frac{1}{x} - \frac{1}{n}}$ For zero events, the “Rule of Three” was applied, yielding a 95% CI of 0 to 3/n.

A possible difference in the distribution of misplaced catheters by subgroup (right vs. left IJV, CVC placed by resident vs. attending, puncture with ultrasound vs. without, ultrasound miniprotocol adherence vs. deviation) was examined by Fisher's exact test.

A convenience sample size based on six months of CVC placement was used. Statistical analyses were performed using R version 4.4.1 (The R Foundation for Statistical Computing, Vienna, Austria), with base packages under the GNU General Public License v2.0. The complete R analysis script is available from the corresponding author upon request.

Open science and data sharing

The study protocol, developed in Microsoft Excel format (Microsoft® Corp., Redmond, WA, USA), and all corresponding ultrasound image files for each study patient are securely stored on the institutional server of Zurich City Hospital. Due to patient confidentiality and local data protection regulations, the full dataset and image files cannot be publicly posted. De-identified versions of the dataset, including image files and the protocol document, can be shared upon reasonable request to the corresponding author, subject to approval by the institutional data protection office.

No external registry entry was made, as this was an internal quality improvement project not requiring prospective registration under Swiss HRA regulations.

## Results

**Figure 1:**
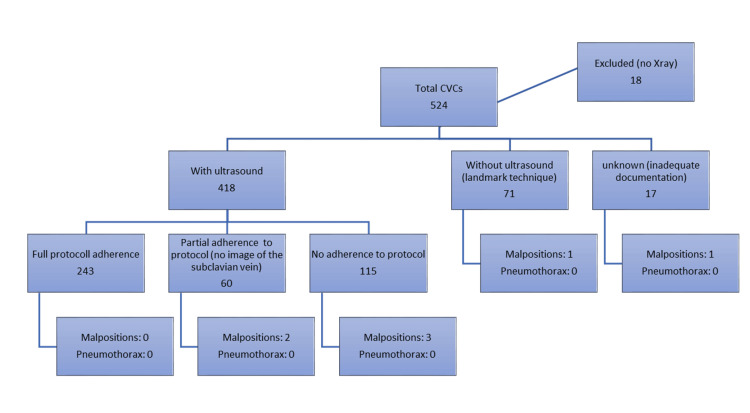
Flow chart for study participants CVC: central venous catheter

During the six-month study period, we documented 524 CVC placements meeting our inclusion criteria. However, 18 were excluded due to a lack of chest X-rays, leaving 506 for analysis. Ultrasound images were acquired in 303 (60%) cases, of which complete adherence to the ultrasound protocol was achieved in 243 cases (48%), while 60 cases (12%) represented partial adherence. A total of 115 (23%) cases were performed with ultrasound but without any acquired images, 71 (14%) cases were performed without ultrasound by the traditional landmark technique, and 17 (3%) had unknown ultrasound status (lacking documentation) (Table 1 and Figure 1).

**Table 1:** Characteristics of CVC insertions (N = 506) CVC: central venous catheter

Variable	n	%
Ultrasound documentation		
Complete adherence (two-step protocol)	243	48
Partial adherence	60	12
Ultrasound used, no images acquired	115	23
Unknown ultrasound status	17	3
Landmark technique (no ultrasound)	71	14
Operator		
Resident	278	55
Attending physician	202	40
Unknown	26	5

Of the 506 CVC insertions, 278 (55%) were performed by residents, 202 (40%) by attending physicians, and 26 (5%) were unknown due to lack of documentation (Table 1). Across all 506 cases, seven malpositions (1.38%, 95% CI: 0.56-2.83) were identified on post-procedural radiographs.

The most significant finding was the complete absence of malpositions in the 243 protocol-adherent cases (0/243, 95% CI: 0-1.2%). In contrast, we identified seven (2.66%, 95% CI: 1.08-5.41) cases of malposition among the 263 partial and non-adherent placements (Table 2).

**Table 2:** Association between protocol adherence and CVC malposition CVC: central venous catheter

Protocol adherence	Total cases (n)	Malpositions, n (%)	Malposition rate (95% CI)	Absolute risk difference (95% CI)	Odds ratio (95% CI)	P-value
Complete adherence	243	0 (0.0)	0.0% (0.0-1.2)	Reference	Reference	-
Partial or non-adherence	263	7 (2.66)	2.66% (1.08-5.41)	-2.66% (-4.74 to -0.86)	∞ (1.18-∞)	0.015
Total	506	7 (1.38)	1.38% (0.56-2.83)	-	-	-

Of the seven cases of malpositions, five did not have any ultrasound images, and in the other two cases, no subclavian images were made (in both cases, the tip was in the subclavian vein). Six of the incorrect placements were in the ipsilateral subclavian vein, and one was in a retrograde cephalad position (Table 3).

**Table 3:** Characteristics of malpositioned catheters (n = 7)

Variable	n
Technique used	
Ultrasound, no images acquired	3
Ultrasound, no subclavian images	2
Landmark technique	1
Unknown	1
Location of malposition	
Ipsilateral subclavian vein	6
Retrograde cephalad	1
Operator	
Attending physician	6
Resident	1

A Fisher's exact test comparing protocol adherence to partial or non-adherence yielded a p-value of 0.015, indicating a statistically significant association between lacking protocol adherence and incorrect catheter placement. Full adherence to the two-step ultrasound protocol was associated with a 2.66% absolute reduction in the risk of CVC malposition compared with partial or non-adherence (absolute risk difference -2.66%, 95% CI: -4.74 to -0.86). Because no malpositions occurred in the adherence group, the estimated odds ratio for malposition in non-adherent versus adherent cases was infinite (95% CI: 1.18-∞), indicating that malposition occurred exclusively in the non-adherence group. Fisher’s exact test demonstrated that this association was statistically significant (p = 0.015). Although the CIs are wide due to the low number of events, the direction and magnitude of the effect suggest that protocol adherence may meaningfully reduce the risk of malposition.

Not enough CVCs were placed in the left IJV (n=17) to statistically compare the effect of the side on misplacement. Interestingly, six of seven malpositions occurred when the CVC was placed by attendings (n = 6) rather than residents (n = 1).

Furthermore, the use of the landmark technique led to one of the seven malpositions, while using ultrasound in the placement led to five of the seven malpositions. In one of the seven malpositions, the ultrasound status is unknown due to a lack of documentation.

Postoperative X-ray reviews identified one pneumothorax in postoperative patients without intrathoracic surgery (n = 172). However, it was on the contralateral, non-punctured side, most likely indicative of another etiology. Given the clinical context, we could not definitively attribute this pneumothorax to the central line placement. This assumption led to a rate of 0% (95% CI: 0-1.7%) (Table 4).

**Table 4:** Procedure-related complications CVC: central venous catheter

Complication	n	Rate (95% CI)
Pneumothorax attributable to CVC	0	0% (0-1.7%)
Pneumothorax contralateral (unrelated)	1	-

## Discussion

Our quality initiative demonstrates that a simple, standardized two-step ultrasound protocol was associated with a low CVC malposition rate of 1.4%, indicating effectiveness in routine practice. Only one questionable pneumothorax was observed. These results support the use of this ultrasound protocol as a quick and reliable alternative to routine post-procedural radiography, with minimal additional effort.

Several studies have validated point-of-care ultrasound (POCUS)-based strategies for post-procedural confirmation of CVC position, yet their day-to-day usability differs markedly. Wen et al. first described a saline‐contrast “bubble test” performed with TTE in 202 patients (219 CVCs). In essence, agitated saline was injected over the tip of the catheter, and if bubbles were promptly observed in the right atrium, the position was assumed to be correct. Only 2/219 (0.9%) catheters were malpositioned, both detected by the test, which took a mean of 3.2 min versus 28.3 min for radiography [10]. Our protocol similarly emphasizes speed and simplicity during placement.

Building on this concept, Weekes et al. prospectively evaluated 151 patients using a protocol of a rapid-atrial-swirl sign (RASS) after saline flush and pleural ultrasound for lung sliding. The study included both internal jugular and subclavian lines and showed that ultrasound correctly confirmed tip location in 147/151 (97%). Chest X-ray found four malpositions, three of which were recognised sonographically. Median examination time was 1.1 min, 20-fold faster than chest X-ray [13].

A larger multicentre trial by Smit et al. applied a three-step protocol for identifying catheter position: bilateral ultrasound examination of the internal jugular and subclavian vein, agitated-saline contrast-enhanced ultrasound, and lung ultrasound in 758 insertions. Malposition occurred in 23/688 analysable studies (3.3%); the protocol showed an ultrasound sensitivity of 70% and specificity of 99%, with ultrasound feasible in 99% of cases. This indicates that complications after CVC insertion are uncommon and that bedside ultrasound is a practical, highly accurate way to detect catheter malposition and procedure-related pneumothorax [14]. In comparison, our two-step protocol avoids additional equipment and steps, making it highly feasible in perioperative practice.

A 2018 systematic review and meta-analysis that pooled 25 studies encompassing 2602 central-line insertions found that bedside ultrasound identified malposition with a pooled sensitivity of 68.2% (95% CI: 54-79) and a specificity of 98.9% (95% CI: 97.8-99.5). Ultrasound images could be obtained in 96.8% of patients, and the examination required a mean of 2.8 min (mean of chest radiography 34.7 min). The rate of malposition was 6.8% and procedure-related pneumothorax was 1.1% [15]. These findings reinforce that rapid ultrasound assessment is accurate, safe, and practical.

Taken together, bubble-enhanced TTE and combined vascular-cardiac-lung ultrasound can identify malpositioned CVCs within minutes and with high specificity. Yet each method carries trade-offs: the bubble test and three-step protocols need extra equipment (agitated-saline syringes, curvilinear probes) and at least one trained assistant, while direct tip visualisation hinges on echocardiographic expertise. In high-turnover or teaching environments, these requirements may limit adoption, and a confirmatory chest radiograph remains prudent whenever image acquisition or interpretation is uncertain.

In contrast, our two-step protocol requires only a linear probe and two brief ultrasound views: one to confirm caudal guidewire direction in the IJV, and another to exclude guidewire presence in the ipsilateral subclavian vein. Both views are attained during CVC placement. In addition to identifying malposition, an incorrect placement can immediately be corrected when visualizing the guidewire in real-time. As ultrasound is generally used for CVC placement anyway, the incremental effort to identify and reduce misplacement with two additional views is minimal. This "bare bones" approach offers a highly feasible alternative that fits seamlessly into routine practice, particularly in anesthesia and perioperative settings. As such, our method prioritizes simplicity and generalizability across clinical settings, leveraging tools and skills already familiar to anesthesia providers.

The finding that most malpositions occurred when attending anesthesiologists rather than residents placed CVC warrants particular attention. This may suggest possible selection bias for difficult cases for attendings or may suggest that even (or particularly) experienced clinicians may benefit from adherence to standardized protocols.

The pneumothorax data further support the safety of uncomplicated jugular CVC placement. Several studies have found that pneumothorax is now an uncommon complication of CVC insertion. McGee and Gould analysed >8,000 landmark-guided cannulations and reported 151 pneumothoraces (≈1.8%); only about 10% of these followed an internal-jugular approach, whereas the vast majority occurred after subclavian access [16]. Another large modern ultrasound-era cohort study by Chui et al. identified just 22 pneumothoraces (0.32%) among 6,875 CVCs, with 86% of events linked to the subclavian rather than the jugular route [17]. These data support the view that pneumothorax is both infrequent overall and disproportionately associated with subclavian placement.

Limitations

This study has several limitations. First, as a quality improvement project conducted at a single tertiary care center, our findings may have limited generalizability to institutions with different patient populations, levels of operator expertise, or ultrasound equipment. However, the described protocol is designed to be simple and uses standard linear probes, which may facilitate its adoption in other settings. Second, the observational design introduces the potential for residual confounding, though it reflects real-world clinical practice. Third, protocol adherence was objectively defined by the saving of ultrasound clips. This method, however, cannot distinguish between true protocol deviation and a potential failure to document an otherwise adherent procedure, which is a key limitation in our analysis of the non-adherence group. A key methodological limitation is our inability to confirm whether guidewire malposition into the subclavian vein occurred and was immediately corrected during the procedure, which would represent an unmeasured successful intervention. Finally, while catheter position was verified by chest X-ray in all analyzed cases, the exclusion of a small number of procedures due to missing imaging and the inherent, though rare, imperfection of radiographic confirmation means that we cannot entirely rule out undetected malpositions.

## Conclusions

This quality improvement project provides encouraging data that a simple, real-time, two-step ultrasound protocol is associated with a lower rate of CVC malposition in the IJV and virtually no pneumothoraces. This finding, together with other studies, suggests that routine post-procedural radiography is unnecessary. Implementation challenges, particularly among experienced providers, highlight the importance of cultural change in adopting new practice standards. Based on our findings, we recommend wider adoption of such ultrasound protocols to improve patient safety, reduce costs, and minimize unnecessary radiation exposure.
